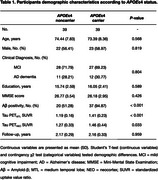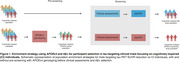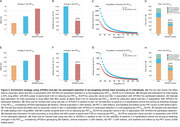# *APOE*ε4 genotyping for populational enrichment of tau‐targeting clinical trials in cognitively impaired individuals

**DOI:** 10.1002/alz70856_107470

**Published:** 2026-01-09

**Authors:** Lucas Bastos Beltrami, João Pedro Ferrari‐Souza, Laura Motter Rosso, Guilherme Povala, Douglas Teixeira Leffa, Firoza Z Lussier, Wagner S. Brum, Cristiano Aguzzoli, Marco De Bastiani, Andrei Bieger, Giovanna Carello‐Collar, Wyllians Vendramini Borelli, Joseph Therriault, Arthur C. Macedo, Nesrine Rahmouni, Diogo O. Souza, Bruna Bellaver, Pamela C.L. Ferreira, Pedro Rosa‐Neto, Tharick A Pascoal, Eduardo R. Zimmer

**Affiliations:** ^1^ Universidade Federal do Rio Grande do Sul, Porto Alegre, Rio Grande do Sul, Brazil; ^2^ Universidade Federal do Rio Grande do Sul, Porto Alegre, RS, Brazil; ^3^ University of Pittsburgh, Pittsburgh, PA, USA; ^4^ Neurology Department, São Lucas Hospital of PUCRS, Porto Alegre, Rio Grande do Sul, Brazil; ^5^ McGill University, Montreal, QC, Canada

## Abstract

**Background:**

The accumulation of tau tangle deposits is a potential target for clinical trials in Alzheimer's disease (AD). It is known that amyloid‐β (Aβ) pathology and the apolipoprotein E ε4 (*APOE*ε4) allele accelerate tau pathology; yet, it is unclear whether assessing both variables could lead to more cost‐effective tau‐targeting trials using tau positron emission tomography (PET) as outcome. Here, we investigated the potential utility of considering *APOE*ε4 carriership for population enrichment in AD trials testing drug effects on tau tangle deposition in cognitively impaired (CI) individuals.

**Method:**

Data was retrieved from the ADNI cohort. We selected CI participants with available clinical assessments, *APOE* genotyping, Aβ PET ([^18^F]Florbetapir or [^18^F]Florbetaben) and tau PET ([^18^F]Flortaucipir) at baseline and a 2‐year follow‐up. Patients with global [^18^F]Florbetapir SUVR >1.11 or [^18^F]Florbetaben SUVR >1.08 were considered Aβ positive (Aβ+). We calculated required sample size and total costs for a hypothetical clinical trial testing a 25% drug effect on reducing tau PET accumulation in the medial temporal lobe (MTL) and neocortex (NEO) with 80% power at alpha level 0.05.

**Result:**

We studied 78 CI individuals over a mean (SD) of 2.16 (0.31) years of follow up (Table 1). Figure 1 displays enrichment strategies based on the use of Aβ positivity alone or *APOE*ε4 carriership associated with Aβ positivity for the selection of patients for a hypothetical tau‐targeting trial in CI individuals. The addition of *APOE*ε4 carriership to Aβ positivity in the population enrichment strategy would notably reduce the required sample sizes (tau PET_MTL_ = 53% and tau PET_NEO_ = 41%), as well as trial costs (tau PET_MTL_ = 55% and tau PET_NEO_ = 44%), compared to using Aβ positivity alone (Figure 2).

**Conclusion:**

Our results support that using *APOE*ε4 genotype together with Aβ positivity for population enrichment to select individuals at higher risk of fast tau accumulation could potentially reduce required sample sizes and costs for tau‐targeting trials focusing on CI individuals. Hence, this may be a cost‐effective strategy.